# Lasing from SOI-Integrated GaAsSb Nanowires via Resonator-Driven Optical Feedback

**DOI:** 10.1021/acs.nanolett.6c02235

**Published:** 2026-07-24

**Authors:** J. Zöllner, C. Doganlar, C. García Marcilla, S. Meder, G. Koblmüller, J. J. Finley

**Affiliations:** ‡ Walter Schottky Institute, TUM School of Natural Sciences, Technical University of Munich, 85748 Garching, Germany; § Institute of Physics and Astronomy, Technical University Berlin, 10623 Berlin, Germany

**Keywords:** III−V nanowire laser, ring resonator, integration on Si, pick-and-place, PL spectroscopy

## Abstract

Silicon photonic integrated circuits critically depend on compact on-chip light sources, for which nanowire (NW) lasers are an attractive solution. However, their practical implementation is often limited by broad emission line widths and poor frequency stability resulting from weak optical feedback. Here, we integrate individual GaAsSb NWs by transfer-printing onto silicon-on-insulator (SOI) racetrack resonators to realize optical feedback at silicon-transparent wavelengths. Finite-difference-time-domain simulations reveal efficient coupling between the hybrid NW–waveguide mode and the fundamental TE resonator mode, with calculated cavity *Q* factors exceeding 10^4^. Experimentally, we observe feedback-induced lasing emission at a low threshold (*P*_th_) of 8.6 ± 1.8 μJ/cm^2^. Compared to identical NW lasers without SOI resonator, the line width is reduced by more than a factor of 4 at 3*P*_th_ and remains stable below 1.8 meV up to 5*P*_th_. Our results demonstrate NW-based light sources on SOI and show that tailored resonator designs enable improved line width control and frequency stabilization.

## Graphical abstract

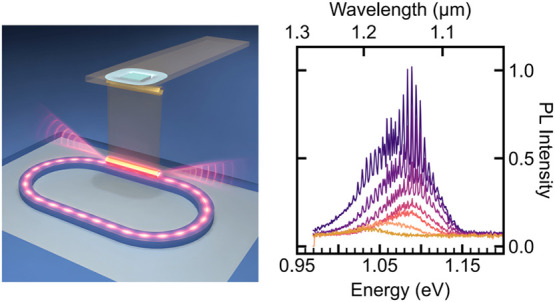

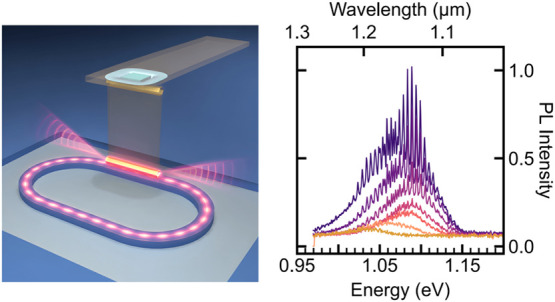

Semiconductor nanowires (NWs) have emerged as promising building blocks for next-generation photonic integrated circuits due to their potential for tailoring material composition, band structure engineering, and integration. For photonics, the inherent geometry of NWs naturally forms an optical Fabry–Pérot cavity, where the NW itself guides optical modes and end facets serve as mirrors enabling optical feedback and lasing operation.^1–7^ At the same time, the nanoscale dimensions provide a small footprint that circumvents strain-induced defect formation when integrated with lattice-mismatched substrates, a critical advantage for monolithic and heterogeneous integration.^8,9^ Several previous studies highlighted these beneficial properties, especially for the integration of III–V NW lasers on silicon (Si) or silicon-on-insulator (SOI) photonic waveguides (WGs) and circuits.^10–16^ Furthermore, their emission wavelengths can be precisely tuned through compositional engineering of the active gain medium, offering flexibility for applications in Si photonics.^17–19^

Despite these advances, existing NW-laser implementations suffer from inherent limitations that constrain their performance, especially the emission line width, stability, modal structure, and frequency control. The relatively short cavity lengths (typically 5–10 μm)^20^ and moderate end-facet reflectivity result in poor geometrical quality factors (Q-factors) on the order of 10^2^, leading to lasing line widths in the high gigahertz range.^21^ Additionally, the optical feedback in conventional NW Fabry–Pérot cavities is primarily determined by the refractive index contrast at the NW–air interface, offering limited tunability for threshold optimization, spectral control, and integration into photonic integrated circuits.

To mitigate these effects, several concepts were proposed that aimed at realizing high-*Q* resonator cavities either from periodic NW arrays or by embedding single NWs into external cavities. Using inverse design, Mauthe et al.^22^ and Takiguchi et al.^23^ demonstrated 1D photonic crystal (PhC) cavities defined by in-plane NW arrays, with some aspects of design flexibility that were limited by geometrical constraints. Another strategy is the hybrid integration of single NWs with PhC cavities to realize ultrahigh Q-factors and very small mode volume, an approach that allows access to stable continuous-wave lasing operation and high-speed modulation in Si-based integrated photonic circuits.^24,25^ Recently, III–V NW lasers were also integrated in-plane onto high-*Q* SiN microring resonators to modify the lasing threshold and line width via evanescent interactions,^26^ building on early demonstrations of NW emitters coupled to lithographically defined PMMA racetrack resonators.^16^ Extending such designs to lasers operating at Si-transparent wavelengths will open very promising concepts for interdevice coupling through waveguide connections and future optical switching investigations in Si photonic integrated circuits. Furthermore, unlike SiN-based platforms, SOI supports carrier doping to enable carrier-induced electro-optical modulation. Additionally, resonator-integrated axially grown p–i–n NW heterojunctions open a direct pathway toward electrically injected NW-based light sources.

In this work, we demonstrate a hybrid III–V NW laser at Si-transparent wavelengths operating through self-driven optical feedback from a SOI racetrack resonator. In this configuration, we utilize GaAsSb-based NWs emitting at ∼1.1–1.2 μm,^27^ integrated onto the straight arm of a SOI racetrack resonator using a precision pick-and-place transfer printing technique. This hybrid architecture enables the high-*Q* Si resonator (*Q*_calc._ = 1.3 × 10^4^) to provide wavelength-selective optical feedback to the NW gain medium, effectively replacing the conventional Fabry–Pérot mechanism with a resonant feedback system that offers enhanced line width control. Thereby, we further demonstrate strong reduction and stabilization in lasing line width under high excitation densities. To elucidate the underlying coupling mechanisms and feedback dynamics of this hybrid system, we complement the experiments with a comprehensive electromagnetic simulation study. Using two-dimensional mode analysis and three-dimensional finite-difference-time-domain (FDTD) simulations, we quantify the hybrid mode profiles, coupling efficiencies, and confinement factors governing NW–WG interaction and directly relate these parameters to the experimentally observed spectral characteristics. This combined approach provides a detailed understanding of how the resonator geometry and NW positioning enable efficient feedback and waveguide-coupled emission in the SOI platform.

Figure 1 presents the core concept and experimental demonstration of our resonator-enhanced NW emission. The device architecture in Figure 1a illustrates our comparative approach: a GaAsSb nanowire (NW1) integrated onto the straight arm of a silicon racetrack resonator and a nominally identical control nanowire (NW2) placed on a simple Si ridge waveguide. The racetrack resonator features 10 μm straight coupling sections with 5-μm-radius semicircular bends, fabricated on a standard SOI platform with a 260-nm-thick and 500-nm-wide Si device layer on a 1-μm-thick SiO_2_ buried oxide layer. The two configurations enable a direct comparison between resonator-coupled and unmodulated NW emission, emphasizing the effect of high-*Q* Si resonator feedback. Parts b and c of Figure 1 show scanning electron microscopy (SEM) images of the fabricated device after NW integration via precision pick-and-place transfer in our custom-built transfer printing setup (Supporting Information S1).^28,29^ As reported in the literature, such heterogeneous pick-and-place integration has recently emerged as a versatile method for creating hybrid NW architectures that decouple growth optimization processes of NWs from the integration process.^30,31^ The overview in Figure 1b captures the complete racetrack resonator structure with the integrated NW, while the magnified view in Figure 1c confirms the precise alignment of the NW along the Si waveguide. The hexagonal NW cross section was consistently aligned to the center of the waveguide within several tens of nanometers, which enables efficient coupling between the NW emission and the guided modes of the resonator.

**Figure 1:**
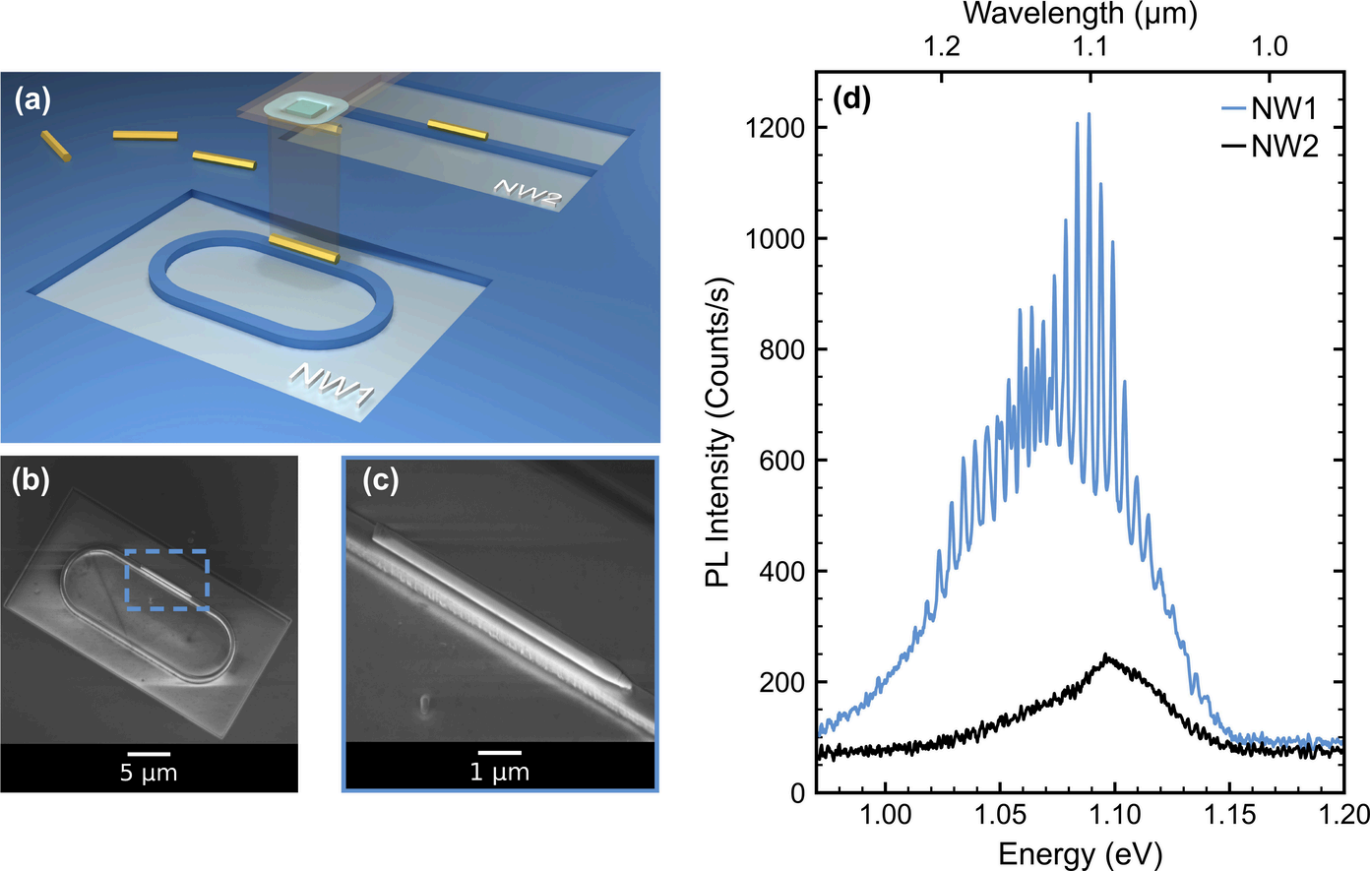
Hybrid NW–resonator platform demonstrating self-driven optical feedback. (a) Schematic representation showing GaAsSb NW integration on two different Si photonic structures: NW1 positioned on a racetrack resonator arm and NW2 on a reference ridge waveguide. (b and c) SEM images of the fabricated racetrack resonator with integrated NW1, where part c shows a magnified view demonstrating precise NW positioning on the Si waveguide. (d) PL spectral comparison at a fixed pump fluence of 35 μJ/cm^2^ revealing distinct spectral modulation in NW1 (blue) characteristic of resonator feedback, in contrast to the emission spectrum of NW2 (black) on the reference waveguide.

The key experimental result is presented in Figure 1d, which compares the photoluminescence (PL) spectra of both configurations under identical excitation conditions. All measurements were conducted at a temperature of 10 K using a Ti:sapphire laser tuned to a wavelength of 780 nm, delivering pulses with a duration of 200 fs at a repetition rate of 82 MHz. The excitation beam was focused onto the NWs with a spot size of 19.9 ± 1.8 μm to ensure homogeneous illumination along the entire NW length. For both spectra shown in Figure 1d, a pump fluence of 35 μJ/cm^2^ was applied. The spectrum of NW1 (blue) exhibits pronounced periodic modulations superimposed on the spontaneous emission from the GaAsSb NW, with peak intensities significantly exceeding the background level. These spectral features directly demonstrate the wavelength-selective amplification induced by the racetrack resonator’s optical feedback. In stark contrast, NW2 (black) displays a smooth, broad emission profile that is found to be intricately linked to the lack of localized photonic modes accessed by the NW. This comparison unambiguously confirms that the observed spectral modulation originates from the resonant coupling between the NW gain medium and the Si racetrack resonator.

To elucidate the physical mechanisms governing light–matter interaction in the hybrid NW–WG platform, we performed a detailed numerical analysis of the supported optical modes and their characteristics. The coupling mechanisms and feedback dynamics in the hybrid NW–resonator system were studied via two-dimensional COMSOL simulations and three-dimensional FDTD simulations, performed using Lumerical. Figure 2a illustrates the hybrid modes supported by the NW–WG structure, which closely resemble the well-known confined modes of hexagonal NWs^32^ as well as the TE and TM guided modes of the Si ridge waveguide. As a first step, we evaluated the coupling efficiency of a guided hybrid mode transitioning into a waveguide mode (Figure 2b, upper panel) as a function of the waveguide width, which was varied from 300 to 900 nm. The variation of the WG dimensions provides insights into the optimum conditions for mode confinement and coupling efficiency. Three coupling configurations were considered: the HE_1,1,a_ hybrid mode coupling to the TM mode, the HE_1,1,b_ mode coupling to the TE mode, and the TE_0,1_ mode coupling to the TE mode. All other combinations exhibit negligible coupling (Supporting Information S2).

**Figure 2:**
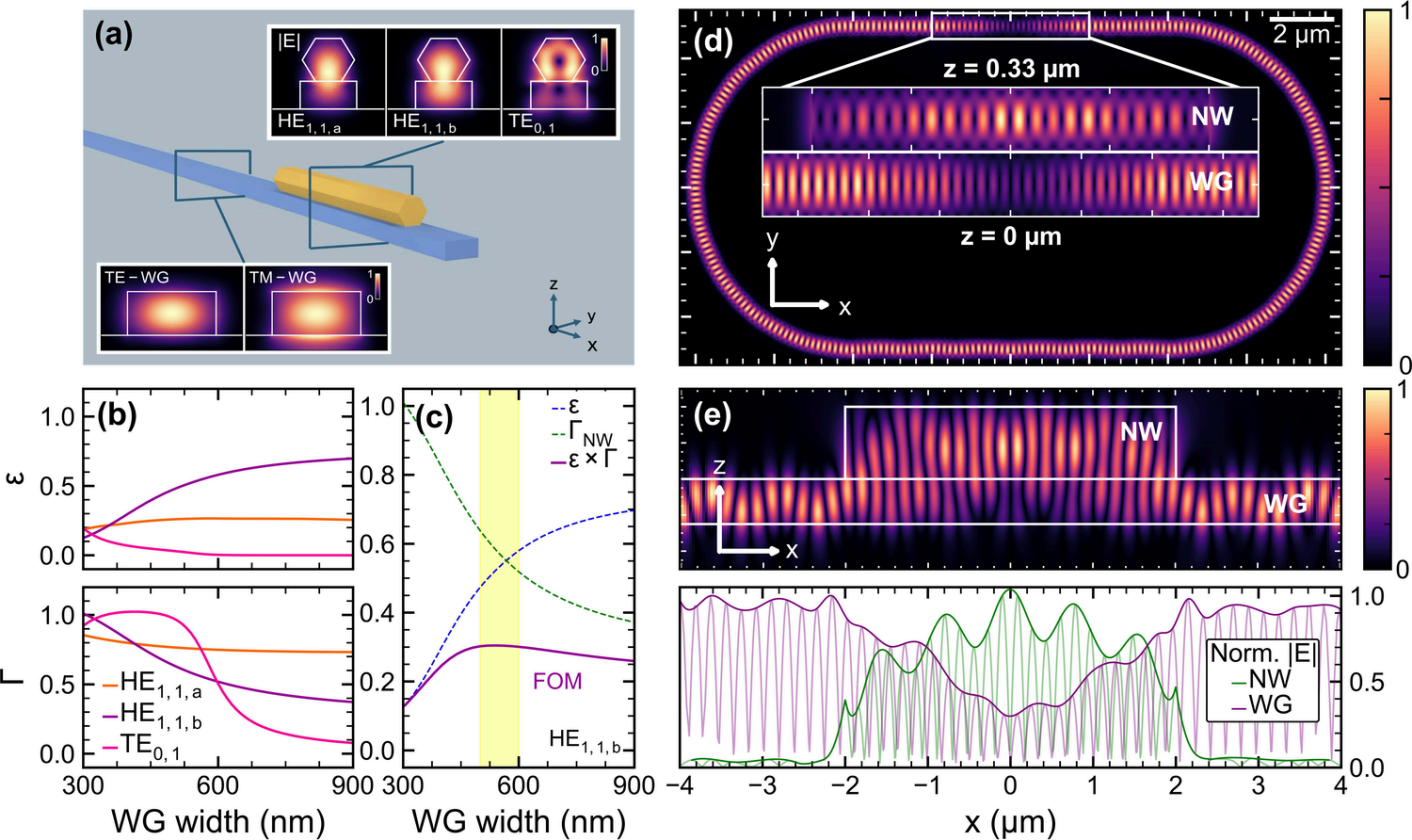
NW–WG coupling simulations. (a) Schematic illustration of the hybrid NW–WG modes and the confined TE/TM guided modes of the silicon ridge waveguide. (b) Upper panel: Coupling efficiency of hybrid modes as a function of the waveguide width for the three mode pairs: HE_1,1,a_ → TM, HE_1,1,b_ → TE, and TE_0,1_ → TE. Lower panel: Confinement factors Γ of the corresponding hybrid modes, illustrating the decreasing overlap with the NW gain medium for increasing waveguide widths. (c) Figure of merit (FOM = εΓ) combining coupling efficiency and confinement, with a maximum for the HE_1,1,b_ → TE configuration at a waveguide width of 500 nm. (d) 3D FDTD simulation of the electric field intensity distribution in the *x*–*y* cross section through the racetrack resonator for the HE_1,1,b_ excitation. Insets: Field distributions for NW and WG cross section. (e) Upper panel: Electric field intensity distribution in the *x*–*z* cross section through the NW–WG coupling region showing strong confinement in the silicon ring and efficient coupling between the NW and the underlying WG. Lower panel: Normalized field distribution in the NW and WG cross section demonstrating quantitative coupling strength.

For both hybrid electric modes, the coupling efficiency increases with increasing WG width. In contrast, the TE_01_ mode exhibits reduced coupling as the WG width increases due to the reduced spatial overlap of the polarization components with the TE WG mode (Supporting Information S2). Furthermore, it is important to note that all hybrid-guided modes already contain a nonnegligible field fraction in the waveguide, meaning that the coupling efficiency alone does not fully capture the performance of the coupled structure. The spatial overlap with the active NW gain medium is equally important, and this is quantified by the confinement factor^33^ given by

$$\Gamma =\frac{2\varepsilon_{0}cn_{a}\iint_{\mathrm{active}}dx\;dy\;{\left|\mathrm{E̅}\right|}^{2}}{\iint dx\;dy\;\left[\mathrm{E̅}{\mathrm{H̅}}^{*}+{\mathrm{E̅}}^{*}\mathrm{H̅}\right]\cdot ẑ}$$

shown in the lower panel of Figure 2b with the refractive index of the homogeneous GaAsSb active region *n*_a_. Here, the integral in the numerator considers the cross section of the homogeneous gain medium, while the denominator integrates over the entire cross section of the NW–WG system. For all three modes, Γ decreases with increasing waveguide width, as a larger fraction of the optical power shifts from the NW into the wider waveguide region. Because an optimal device requires both strong confinement in the gain medium and efficient coupling into the Si waveguide, we introduce FOM = εΓ, shown in Figure 2c. The FOM reaches its maximum for the HE_1,1,b_ hybrid mode coupling to the TE waveguide mode at a waveguide width of approximately 500 nm. This justifies the choice of our experimental WG width used throughout this study.

This result further motivated full 3D FDTD simulations of the proposed racetrack resonator using the HE_1,1,b_ mode as the injected source. Spectral analysis of the coupled system, obtained through Fourier transformation of time-monitored signals within the NW region, reveals multiple discrete resonance peaks (Supporting Information S3). These sharp resonances correspond to different longitudinal modes of the ring cavity that satisfy the resonance condition. A resonant mode at an energy of 1.12 eV, close to the maximum gain of our GaAsSb NWs, was chosen to calculate the electric field distribution viewed from different perspectives. The simulated in-plane (*x*–*y*) electric-field distribution (Figure 2d) reveals strong optical confinement in the silicon racetrack resonator with the characteristic standing-wave pattern of the circulating TE mode. The inset cross section through the coupling region highlights the vertical mode overlap between the NW and waveguide. The hexagonal GaAsSb NW is positioned directly on top of the Si WG, enabling efficient evanescent coupling between the hybrid NW mode and the fundamental TE waveguide mode of the resonator. The vertical field profile (Figure 2e, upper panel) confirms that the electric field extends continuously from the waveguide into the NW, demonstrating strong penetration into the active region, which is quantified in the lower panel of Figure 2e. Here we find that more than 50% of the TE WG mode is transferred to the NW in the center of the coupling region. The correlation between the coupling efficiency, confinement factor, and spatial field distributions validates our design choices and confirms the self-driven optical feedback scheme. Recent work from Yi et al. has demonstrated microring-assisted NW lasing on a silicon nitride photonics platform at room temperature,^26^ highlighting the general potential of resonator-mediated feedback for stabilizing NW-type emitters. In contrast, our approach establishes a hybrid GaAsSb NW laser directly integrated on the SOI platform, where the NW couples to the fundamental TE mode of a Si waveguide resonator operating in the Si-transparent near-infrared regime. Importantly, the NW is positioned on top of a prefabricated photonic component, enabling a transfer-based integration approach that supports postfabrication assembly on Si photonic structures in a very flexible manner. With a higher refractive index of Si compared to SiN, our device offers a footprint as small as 10 μm × 15 μm, which is comparable to state-of-the-art WG-coupled NW-induced PhC cavity designs.^23^

Having established the geometric configuration and optical mode engineering underlying the efficient NW–resonator coupling, we now present the experimental findings of this hybrid integration under optical pumping. In particular, we investigate how the strong evanescent interaction and self-driven optical feedback discussed in relation to Figure 2 is displayed in the emission characteristics of individual GaAsSb NWs emitting at Si-transparent wavelengths. By directly comparing the pump-fluence-dependent microphotoluminescence (μ-PL) response of NWs coupled to a racetrack resonator with that of reference standalone NWs lying on sapphire, we isolate the role of resonator-induced feedback shaping the spectral characteristics.

Figure 3 illustrates this direct comparison of the two different integration cases, highlighting the impact of resonator-induced optical feedback combined with efficient waveguide coupling. First, Figure 3a depicts the recorded spectra of a single GaAsSb NW integrated on a racetrack resonator under pulsed excitation at a temperature of 10 K. At low excitation levels, the emission exhibits a smooth and broad spectrum spanning approximately between 1.0 and 1.15 eV (∼1.1–1.2 μm), consistent with spontaneous emission from bulk GaAsSb NWs.^26^ With increasing pump fluence (0.8 μJ/cm^2^), pronounced spectral modulations start to emerge, indicating the onset of optical feedback induced by the photonic resonator. At elevated pump fluences (>9.1 μJ/cm^2^), equally spaced peaks dominate the emission spectrum, arising from efficient coupling of the NW emission into the propagating modes of the racetrack resonator. The dominant mode is highlighted by a purple arrow. The spacing between adjacent resonances (Δ*E* = 5.2 meV) is characteristic of the extended optical path length (*L*_RR_ ∼ 51.4 μm) of the racetrack resonator compared to the standalone NW (*L*_NW_ ∼ 5 μm), confirming that they originate from the coupled system. This observation demonstrates the anticipated optical feedback, validating the central design goal of the integrated device. The inset shows a microscope image of the device under laser excitation, where both the emitted light from the NW and the scattered light coupled to the resonator are visible. The input–output characteristics of the dominant resonant peak are shown in Figure 3b. The peak intensity (purple) is plotted as a function of pump fluence alongside the averaged intensity of the two adjacent spectral minima (blue). A clear nonlinear increase in the peak intensity marks the onset (8.6 ± 1.8 μJ/cm^2^) of stimulated emission. At high pump fluences (>30 μJ/cm^2^), a reduction in the emission intensity is found, an observation that we attribute to heating effects. The lasing threshold is determined from linear fits to the spontaneous emission regime below the threshold (blue line) and the stimulated emission regime above the threshold (purple line), with the intersection defining the threshold pump fluence. Figure 3c further demonstrates lasing behavior by presenting the input–output characteristics on a log–log scale. The resulting S-shaped curve is characteristic of the transition from spontaneous to stimulated emission. Starting with a linear spontaneous emission regime the extracted slope increases at the threshold and subsequently approaches unity at higher pump fluences, consistent with the expected behavior of a lasing system. The less pronounced S-like characteristic in the LL curve of the resonator device compared to typical standalone NWs^6,11^ is attributed to strong mode competition (Supporting Information S3). Additional analysis presented in Supporting Information S4 provides further evidence for resonator-mediated lasing. To assess the reproducibility of the observed behavior, we performed pump-fluence-dependent μ-PL measurements on multiple nominally identical GaAsSb NW devices integrated on racetrack resonators and compared them to standalone NWs on sapphire. Across all measured resonator-coupled NWs (Supporting Information S5), we consistently observe the emergence of resonator-mode modulation at elevated excitation densities, accompanied by comparable lasing thresholds ranging between 8.6 and 15.5 μJ/cm^2^. Importantly, in all cases, the resonator-integrated configuration exhibits enhanced spectral stability at higher pump fluences: across three additional devices the line width remains nearly constant at ∼2 meV above the threshold (Supporting Information S5). These statistics confirm that the reduced threshold and improved frequency stability are intrinsic features of the ring-resonator-coupled geometry rather than a single-device effect.

**Figure 3:**
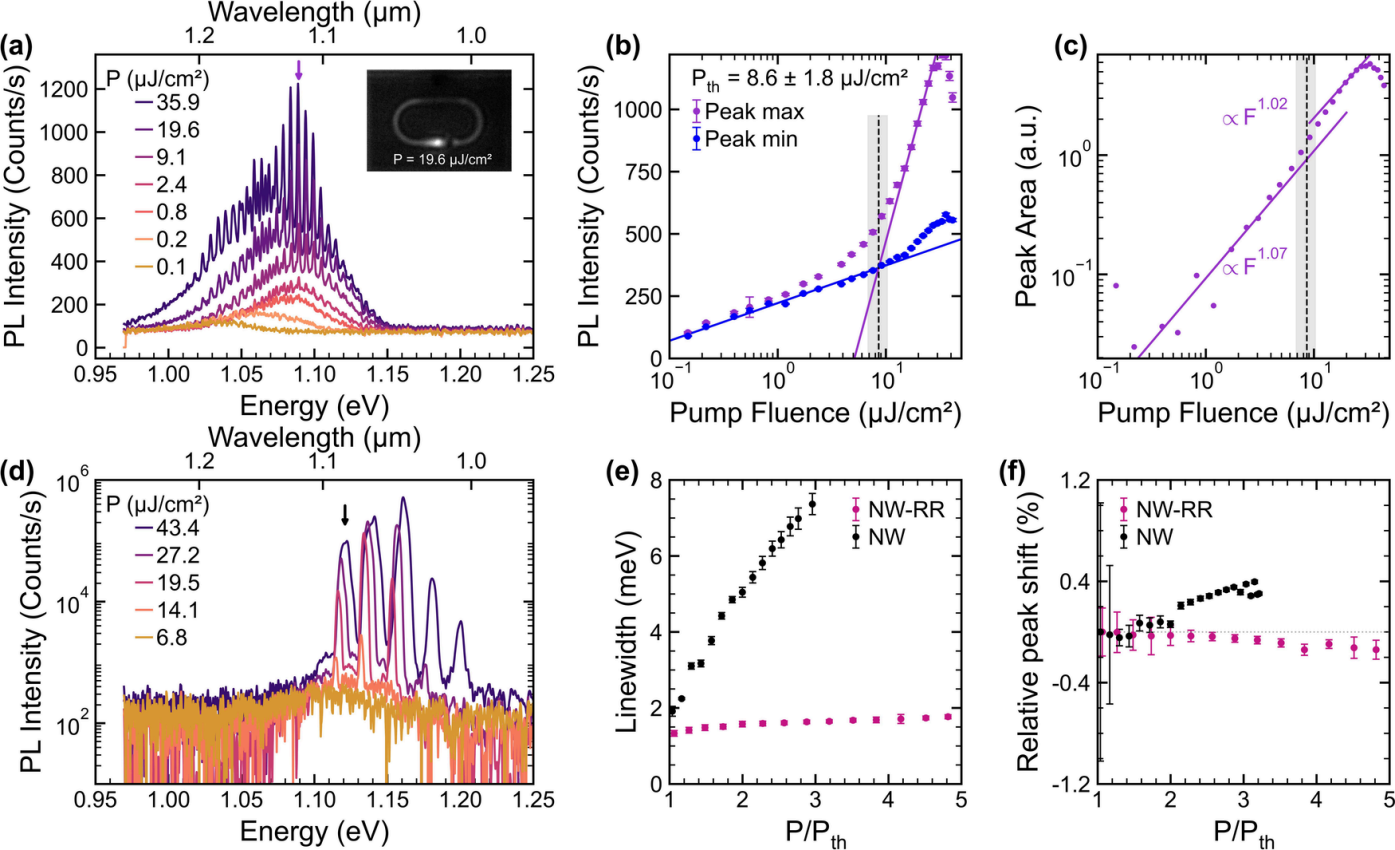
Comparison between pump-fluence-dependent μ-PL measurements of an individual GaAsSb NW on sapphire versus on a racetrack resonator. (a) Spectra of a GaAsSb NW coupled to a racetrack resonator showing lasing emission at 10 K. The dominant lasing peak is indicated by the purple arrow. Inset: Microscope image of an optically excited NW–resonator system. (b) Input–output characteristics of the dominant peak (purple) and adjacent spectral minima (blue), with the lasing threshold (dashed line) extracted from linear fits. (c) Log–log input–output characteristics showing the typical S-shaped curve of a laser. (d) Spectra of a standalone GaAsSb NW on sapphire at 10 K, where optical feedback arises from NW facets. (e) Line width comparison between standalone NW (black) and resonator-coupled NW (magenta) showing reduced line width and enhanced stability for the resonator system. (f) Corresponding relative lasing peak shift, normalized to the respective peak energy at *P* = *P*_th_, highlighting improved spectral stability for the ring-resonator-coupled device compared to the standalone NW.

For comparison, Figure 3d shows pump-fluence-dependent μ-PL spectra of an equivalent standalone GaAsSb NW lying on a sapphire substrate under the same excitation conditions. In this configuration, optical feedback is provided solely by reflections at the NW end-facets, resulting in a more than 20 times lower calculated geometrical Q-factor compared to the resonator-based system. Nonetheless, a transition to lasing behavior is observed with increasing pumping. Parts e and f of Figure 3 quantitatively compare the measured line widths and frequency stability of both systems. As shown in Figure 3e, the emission line width narrows sharply at the threshold, reaching 2.0 meV for the standalone NW (black) and 1.3 meV for the ring-resonator-coupled NW (magenta). At elevated pump fluences, the resonator-integrated system exhibits significantly enhanced spectral stability. At 3 times the threshold pump fluence, the line width increases by only ∼20%, whereas the standalone NW shows more than 300% broadening from 2.0 to 7.3 meV under these conditions. Consistently, the improved spectral stability is also evident in Figure 3f, which plots the relative shift of the dominant lasing peak above the threshold with respect to its spectral position at *P* = *P*_th_. At an excitation fluence of 3*P*_th_, the standalone NW on sapphire exhibits a pronounced blue-shift of approximately 0.4%, whereas the resonator-coupled NW shows only a minor red-shift of roughly 0.05%. The larger peak shift observed for the standalone NW is attributed to a carrier-induced decrease of the refractive index^34^ because the entire cavity is formed by the active NW material. In contrast, the effective cavity of the resonator-integrated device is largely defined by the passive Si racetrack resonator, which mitigates effects arising from refractive index variations of the active medium and thereby stabilizes the lasing wavelength. The slight red-shift observed in the resonator-coupled case is consistent with residual thermal heating of the device at elevated pump fluences, leading to a temperature-induced increase of the effective refractive index for both the active NW and passive resonator material. While our observations regarding lower lasing thresholds, narrower line width and enhanced frequency stability are consistent with the recently published findings from Yi et al.,^26^ our approach offers several decisive advantages. First, the higher refractive index of Si compared to SiN yields a significantly more compact device footprint, comparable to state-of-the-art PhC cavity designs.^24^ Second, operation at Si-transparent wavelengths enables direct routing of the laser output through established SOI photonic networks, a capability unavailable on SiN platforms at these wavelengths. Third, the postfabrication pick-and-place integration onto prefabricated SOI components offers a more deterministic and scalable assembly route. Fourth, we envision the demonstrated platform as a promising pathway toward electrically injected silicon-integrated NW lasers. The exposed, evanescently coupled NW geometry is compatible with established axial p–i–n NW designs and could enable direct electrical contacting of the gain medium while maintaining efficient coupling to the silicon resonator. This capability represents a significant advantage over buried NW architectures and may facilitate the realization of fully integrated electrically driven light sources on the silicon photonics platform. More broadly, the hybrid NW–resonator platform may enable future studies of coupled-cavity phenomena, including engineered mode interactions, coherent interference effects, and active-passive photonic systems based on integrated silicon photonics.

In summary, we demonstrated a hybrid GaAsSb NW–silicon racetrack resonator platform that enables resonator-mediated optical feedback and efficient evanescent coupling into guided TE WG modes. The integrated devices exhibit pronounced spectral modulation and the emergence of regularly spaced narrow peaks, evidencing cavity-enhanced amplification and stimulated emission. Lasing is consistently observed across multiple integrated devices with thresholds of 8.6–15.5 μJ/cm^2^ and line widths remaining near 2 meV, even well above the threshold. The best-performing device exhibits a threshold of 8.6 ± 1.8 μJ/cm^2^, a line width of 1.3 meV at the threshold, and a relative peak shift of less than 0.1% up to *P* = 3*P*_th_. Compared to standalone GaAsSb NWs on sapphire, this platform enables reduced lasing thresholds and strongly improved line width and emission-energy stability. These results establish low-threshold lasing from individual GaAsSb NWs directly onto a silicon photonics platform, providing a compact and scalable route toward nanoscale near-infrared light sources that are integrable into photonic integrated circuits. Future work will focus on deterministic mode control and cavity engineering to enable robust single-mode operation. Looking further ahead, the CMOS-compatible SOI-integrated design demonstrated in this work provides a concrete pathway toward electrically injected NW laser devices.

## Supplementary Material



## Notes

Details of device fabrication and transfer printing technique, FDTD simulations of mode profiles, Purcell and Q-factor calculation, and PL spectroscopy of additional NW lasers (PDF)
