# Supplementary material for: Lasing from SOI-Integrated GaAsSb Nanowires via Resonator-Driven Optical Feedback
Source: Nano Lett. 2026 Jul 24;26(30):9888–94. doi: 10.1021/acs.nanolett.6c02235 (PMC13450476; doi:10.1021/acs.nanolett.6c02235)
Supplement: Supplementary file 1 [file nl6c02235_si_001.pdf]

Supporting Information for

Lasing from SOI-integrated GaAsSb nanowires via resonator-driven optical feedback

J. Zöllner[1], C. Doganlar,[1,2*], C. García Marcilla[1], S. Meder[1], G. Koblmüller[2] and J. J. Finley[1]*

[1]Walter Schottky Institute, TUM School of Natural Sciences, Technical University of Munich, 85748 Garching, Germany

[2]Institute of Physics and Astronomy, Technical University Berlin, 10623 Berlin, Germany

*Corresponding authors.

In this work, both corresponding authors contributed equally.

E-mail: jona.zoellner@tum.de (J.Z.); cem.doganlar@tum.de (C.D.)

S1. Fabrication and Transfer Printing

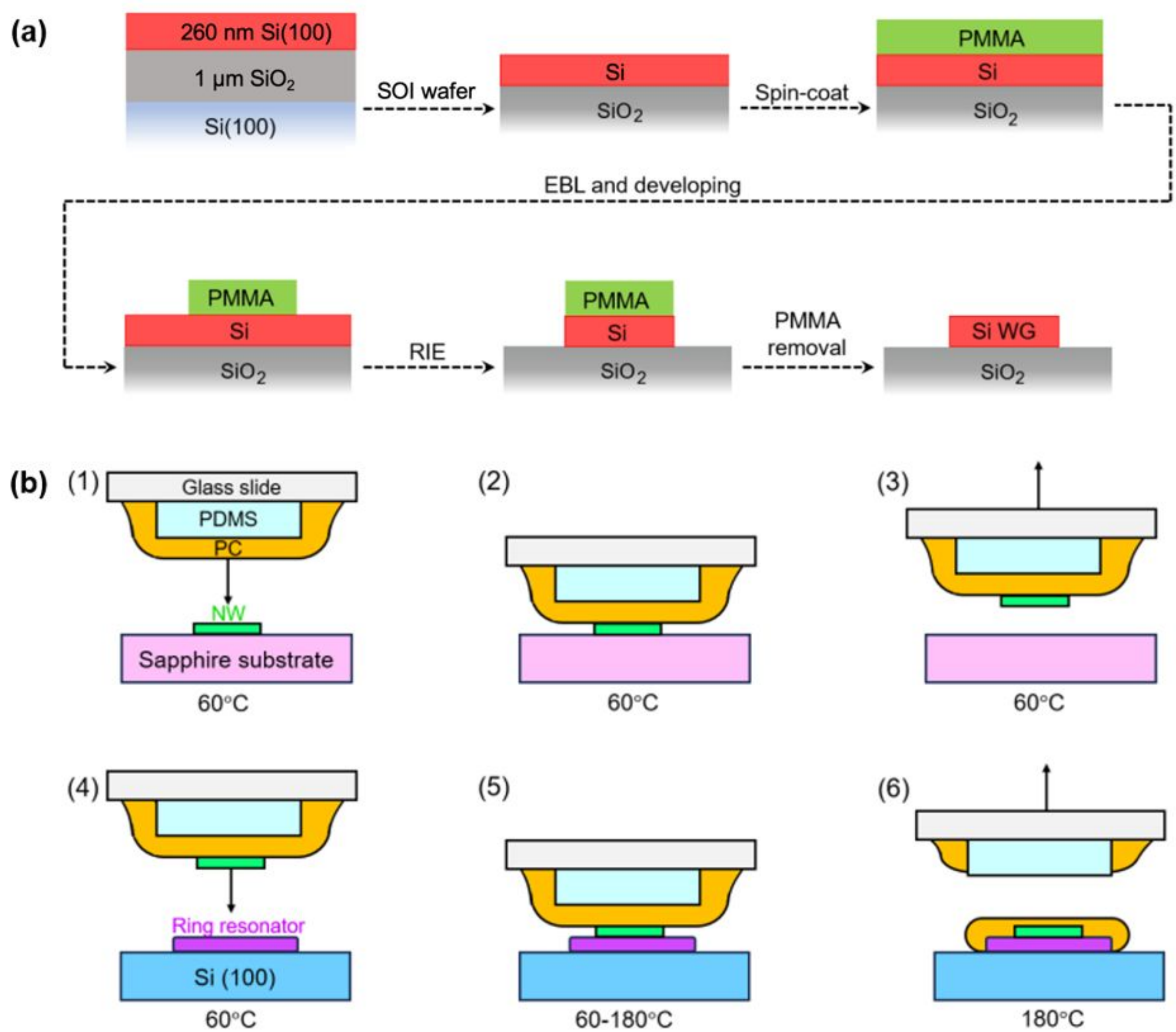

Figure S1: Fabrication flow diagram of the SOI WG platform and NW transfer printing technique. (a) Fabrication flow for defining SOI waveguides. The 260 nm thick Si device layer is patterned using PMMA and EBL, and subsequently etched to form the silicon waveguide structures on SiO_2. (b) Schematic of the transfer-printing process used to deterministically place GaAsSb NWs from a sapphire substrate onto the SOI racetrack resonator using a PDMS/PC stamp, followed by thermal release and PC removal.

Figure S1a illustrates the main fabrication steps used to prepare the silicon-on-insulator (SOI) racetrack resonator. The process starts from a SOI wafer consisting of a 260 nm thick Si(100) device layer on top of a 1 μm buried SiO_2 layer, supported by a thick Si(100) handle wafer. First,

a PMMA resist layer is deposited by spin-coating. This resist serves as an electron-beam lithography (EBL) mask, allowing the waveguide pattern to be written with high precision. Following EBL exposure and development, the remaining PMMA defines the waveguide layout. The pattern is then transferred into the silicon layer using reactive ion etching (RIE), selectively removing the exposed silicon while leaving the masked regions intact. Finally, the PMMA is removed, resulting in the final silicon waveguide structures on the SiO_2 buried oxide layer. **Figure S1**b illustrates the deterministic transfer-printing procedure used to integrate individual GaAsSb NWs onto the silicon racetrack resonator. The nanowires are initially dispersed on a sapphire substrate (where they were measured for reference) and are subsequently picked up using a transparent stamp consisting of a PDMS support layer coated with a thin polycarbonate (PC) sacrificial film. The pickup step is performed at 60 °C, enabling reliable adhesion of the nanowires to the PC layer. After pickup, the stamp is aligned to the target silicon photonic circuit and brought into contact with the straight arm of the racetrack resonator, again at 60 °C. This allows controlled placement of the nanowire at the desired position on the resonator. In our experiments, the nanowires were consistently aligned to the center of the waveguide within several tens of nanometers. Following placement, the sample is heated to 180 °C, i.e., above the glass transition temperature of PC, which enables release of the sacrificial layer from the stamp and leaves the nanowire positioned on the resonator. Finally, the PC layer is removed by dissolution in chloroform. Notably, the nanowires remain attached to the silicon surface and are not displaced during the solvent-based removal process, indicating strong adhesion between the nanowire and the device surface. Importantly, transfer printing does not measurably degrade the optical performance: test measurements performed on the same nanowire before and after transfer show no detectable change in lasing threshold, consistent with previous reports by Jevtics et al.[1]

S2. Polarization Resolved Mode Profiles

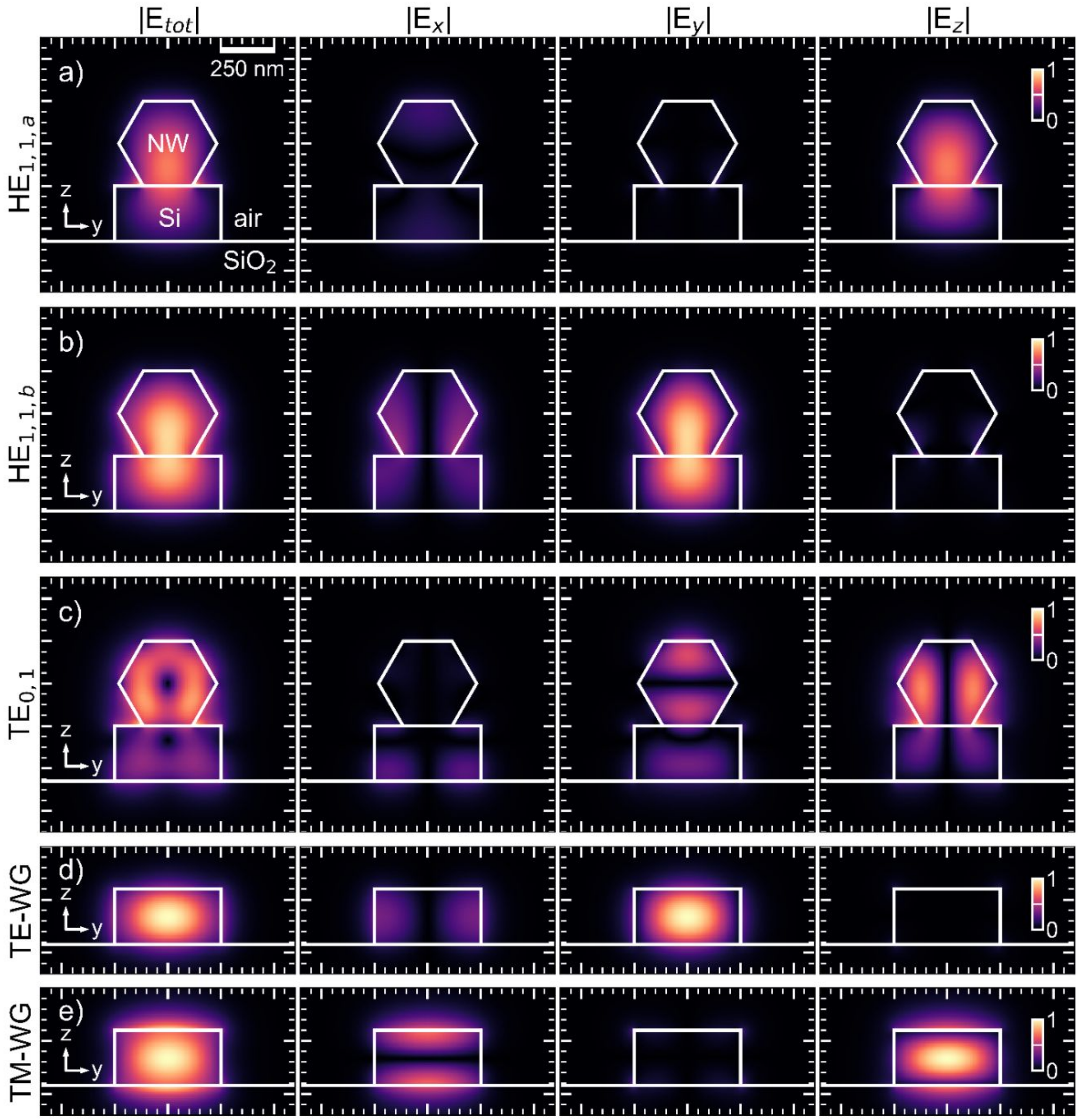

Figure S2: Simulated guided-mode field distributions of the NW-WG hybrid structure and standalone WG. (a–c) Calculated electric-field intensity profiles of the lowest-order hybrid eigenmodes supported by the combined hexagonal NW and rectangular SOI waveguide cross section. The white outlines indicate the simulated geometry consisting of the GaAsSb NW on top of the Si WG with SiO_2 bottom cladding surrounded by air/vacuum. The scale bar corresponds to 250 nm. Shown are the total field magnitude $|E_{tot}|$ and the individual Cartesian field components $|E_x|$, $|E_y|$, and $|E_z|$. Panels (a) and (b) correspond to two $HE_{1,1}$-like hybrid modes ($HE_{1,1,a}$ and $HE_{1,1,b}$), while (c) shows the $TE_{0,1}$-like mode. (d,e) Reference field distributions of the fundamental TE and TM waveguide modes of the bare SOI waveguide without the NW. All field maps are normalized to their respective $|E_{tot}|$.

Figure S2 visualizes the simulated optical eigenmodes of the hybrid system formed by the GaAsSb nanowire placed on top of the silicon waveguide. The field maps reveal how the supported modes and their polarization components are spatially distributed, i.e., how strongly the optical field overlaps with the nanowire versus being confined in the SOI waveguide. The two lowest-order modes $HE_{1,1,a}$ and $HE_{1,1,b}$ exhibit strong intensity localization within the NW cross section while simultaneously maintaining appreciable overlap with the underlying silicon waveguide. This confirms that the nanowire does not act as an isolated cavity but becomes optically coupled to the silicon photonic circuit. The $TE_{0,1}$-like mode shows a similar pattern as compared to the $TE_{0,1}$ guided modes typically found in conventional standalone NWs. Finally, the bottom panels show the fundamental TE and TM modes of the bare silicon waveguide, serving as a reference for comparison and highlighting how the nanowire perturbs and hybridizes the guided-wave modes. Overall, the simulations demonstrate that the hybrid structure supports low-order modes with significant overlap between the nanowire gain region and the SOI waveguide mode, enabling efficient coupling of nanowire emission into the silicon resonator circuit.

S3. FDTD Spectra, Q-factor and Purcell-factor Calculations

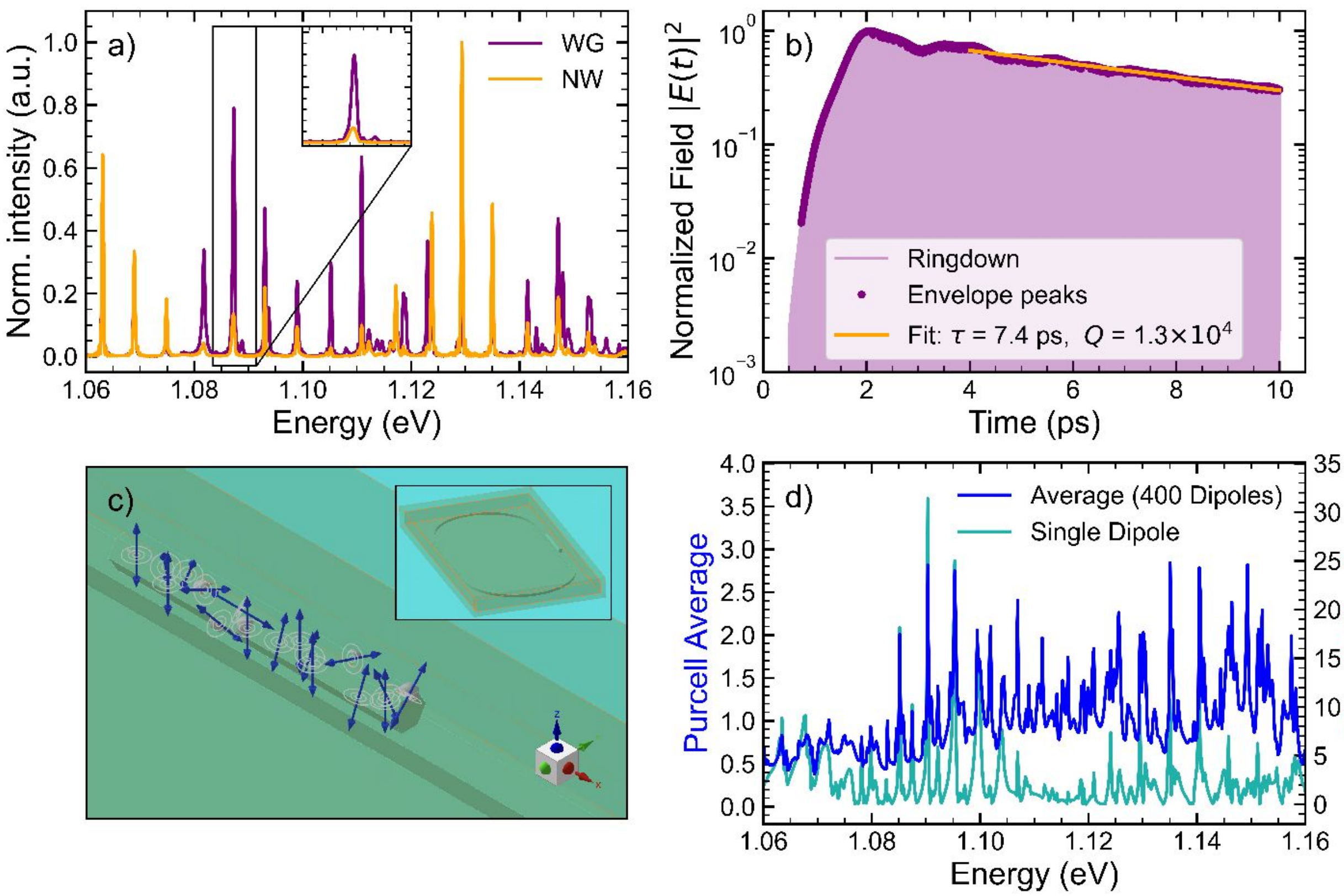

Figure S3: FDTD analysis of NW-WG coupling and cavity enhancement. (a) Fourier-transformed spectra recorded at time monitors inside the nanowire and silicon waveguide under $HE_{1,1,b}$ mode excitation. (b) Ringdown analysis of a selected resonance used to extract photon lifetime and cavity Q-factor. (c) FDTD simulation geometry for Purcell-factor calculations using 400 randomly distributed and oriented dipole emitters inside the NW (few dipoles shown for reference). (d) Spectrally resolved Purcell enhancement for the ensemble-averaged dipoles and for a single optimally positioned dipole.

Figure S3 summarizes the FDTD simulations used to quantify optical coupling between the GaAsSb nanowire and the silicon racetrack resonator, as well as the resulting cavity-induced enhancement of spontaneous emission. In **Figure S3**a, the hybrid structure is excited by injecting the $HE_{1,1,b}$ -like nanowire mode. Time monitors are placed inside the NW and in the silicon waveguide (WG), and the recorded time-domain fields are Fourier transformed to obtain the corresponding spectra. Distinct resonances appear at identical energies in both the NW (orange)

and WG (purple) spectra. This simultaneous appearance confirms that the resonant modes are not confined to only one component but are hybridized across the NW–WG system, demonstrating strong spatial overlap and efficient optical coupling. The inset emphasizes a representative resonance, showing that the spectral feature is clearly present in both monitoring regions. To quantify the resonator performance, **Figure S3**b presents a cavity ringdown analysis for the resonance highlighted in the inset of panel (a). After excitation, the temporal decay of the normalized electric field intensity $|E(t)|^2$ is evaluated. The envelope maxima are extracted (purple markers) and fitted with an exponential decay, appearing linear on the semilogarithmic scale (orange fit curve). From this fit, a photon lifetime of $\tau = 7.4$ ps is obtained, corresponding to a calculated quality factor of $Q = 1.3 \times 10^4$. This confirms that the hybrid system supports high-Q resonances enabling the observed feedback-assisted lasing. While the analysis in (a,b) probes coupling under guided-mode excitation, **Figure S3**c investigates the enhancement of spontaneous emission into the resonator modes. Here, the nanowire is excited using a set of randomly oriented dipole point sources distributed uniformly throughout the entire nanowire volume. Each dipole is assigned a random phase and orientation to mimic an incoherent ensemble emitter distribution. A total of 400 dipoles is used in the simulation (only a small number is shown schematically for clarity). This approach provides a realistic estimate of the average emission enhancement expected from the full gain volume. The resulting Purcell-factor calculations are shown in **Fig. S3**d. The dark-blue curve represents the ensemble-averaged Purcell factor obtained by averaging over all 400 dipoles (left axis). In addition, the light-blue curve shows a single dipole located at an optimal position and orientation, exhibiting high Purcell enhancement up to 32 (right axis) at comparable energies with respect to our measured dominant lasing emission. This implies that a significant fraction of spontaneous emission is funneled into the lasing modes, which increases mode

competition and reduces the sharpness of the classical "S-shaped" input–output curve in nanolasers.[2] In addition, since multiple resonator modes share a common carrier reservoir, gain clamping is expected to be weaker than in an isolated single-mode laser, further contributing to the comparatively gradual threshold transition observed experimentally. This provides a consistent explanation for the comparatively smooth lasing transition observed experimentally.

S4. Micro-Photoluminescence (μ-PL) Spectroscopy

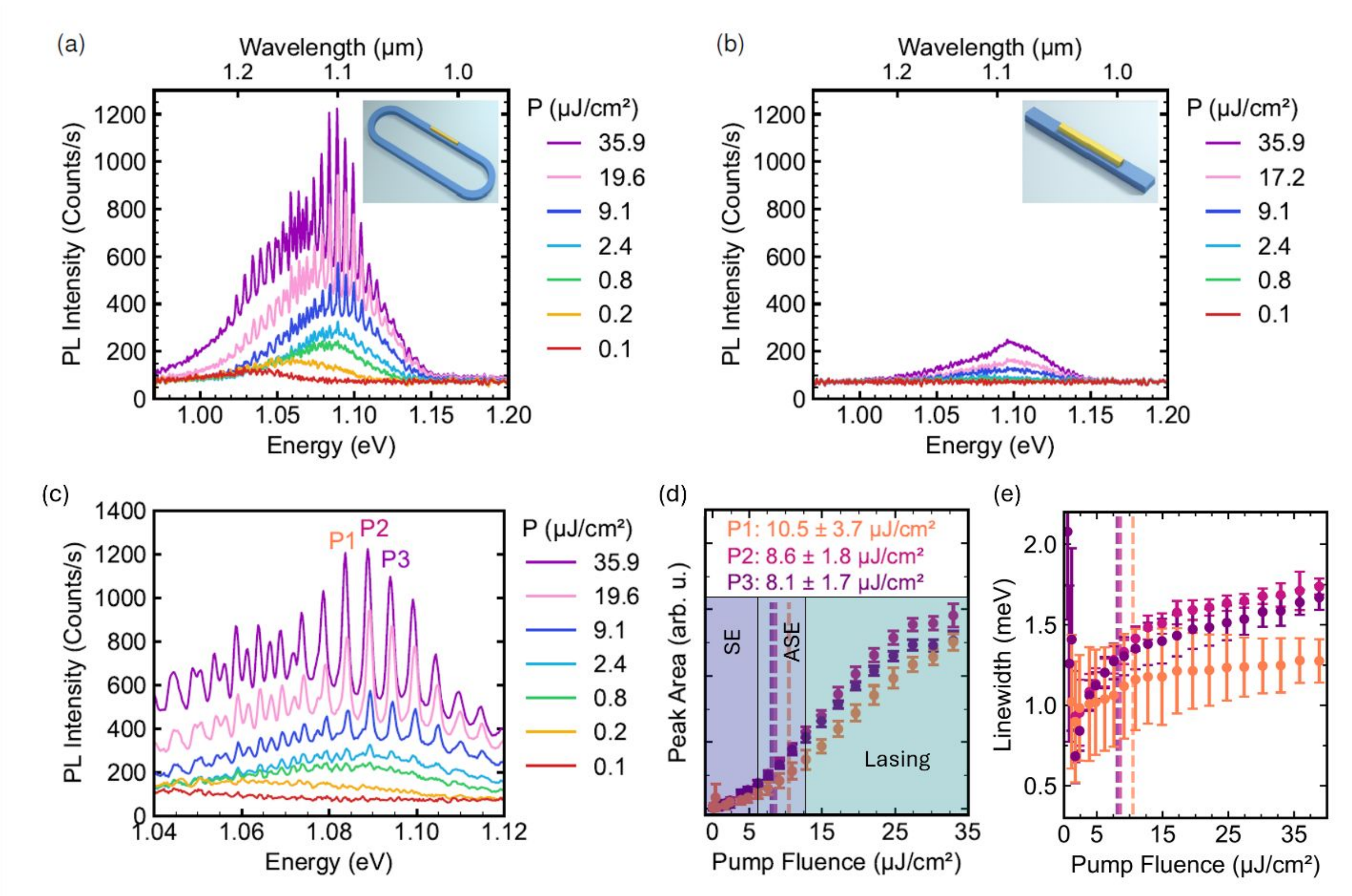

Figure S4. Additional evidence supporting resonator-mediated lasing. (a) Pump-fluence-dependent PL of a nanowire integrated on a silicon racetrack resonator, showing the emergence of narrow resonator modes above threshold. (b) Corresponding spectra from an identical nanowire coupled to a straight silicon waveguide without resonator feedback, exhibiting only broadband emission. (c) Enlarged view of the lasing region highlighting the three dominant resonator modes (P1–P3). (d) Peak area of modes P1–P3 as a function of pump fluence, revealing distinct threshold fluences and indicating transitions from spontaneous emission (SE) to amplified spontaneous emission (ASE) and lasing. (e) Linewidth evolution of modes P1–P3 with increasing pump fluence, showing linewidth narrowing and subsequent stabilization above threshold.

To further assess the origin of the observed emission, additional measurements were performed on both nanowire–resonator devices and control structures consisting of nanowires integrated on straight silicon waveguides without resonator feedback (**Figure S4**).

Figures S4a and **S4**b compare the pump-fluence-dependent emission spectra of the two systems. While both devices exhibit an overall increase in emission intensity with increasing excitation density, only the resonator-coupled device develops narrow, equally spaced resonances with an energy spacing of approximately 5.2 meV, consistent with the expected free spectral range of the silicon racetrack resonator. In contrast, the straight-waveguide control device exhibits only broadband emission throughout the investigated excitation range. This comparison demonstrates that the resonator provides the optical feedback responsible for mode formation, whereas the nanowire Fabry–Pérot cavity alone does not produce comparable spectral features.

At low excitation densities, the spectra of the resonator-coupled devices remain broad and largely unmodulated. Distinct resonator modes emerge only above a characteristic pump fluence and subsequently exhibit nonlinear intensity growth and linewidth narrowing, indicating a gain-dependent transition in the emission dynamics.

A closer inspection of the three dominant resonances (P1–P3) is shown in **Figure S4**c. The corresponding pump-dependent peak areas are plotted in **Figure S4**d, yielding threshold fluences of 8.1 ± 1.7, 8.6 ± 1.8, and 10.5 ± 3.7 μJ/cm², respectively. The intensity evolution exhibits three characteristic regimes: an initial spontaneous-emission regime, a super-linear increase around threshold, and a subsequent approximately linear increase at higher excitation densities. Such behavior is consistent with the onset of stimulated emission and gain clamping within the resonator modes. Furthermore, resonances located farther from the gain maximum do not exhibit

comparably pronounced threshold behavior, consistent with the reduced modal gain available at these energies.

Additional evidence is provided by the linewidth evolution shown in **Figure S4**e. The dominant resonator modes exhibit a pronounced linewidth reduction followed by linewidth stabilization above threshold. The coincidence of linewidth narrowing, nonlinear intensity increase, and resonator-mode selection at comparable pump fluences is consistent with a transition from spontaneous to stimulated emission.

Taken together, the emergence of resonator modes only above a critical excitation density, the absence of comparable features in the straight-waveguide control device, the mode-dependent threshold behavior, and the threshold-associated linewidth evolution support the interpretation of the observed emission as resonator-mediated lasing.

S5. Micro-Photoluminescence (μ-PL) Spectroscopy

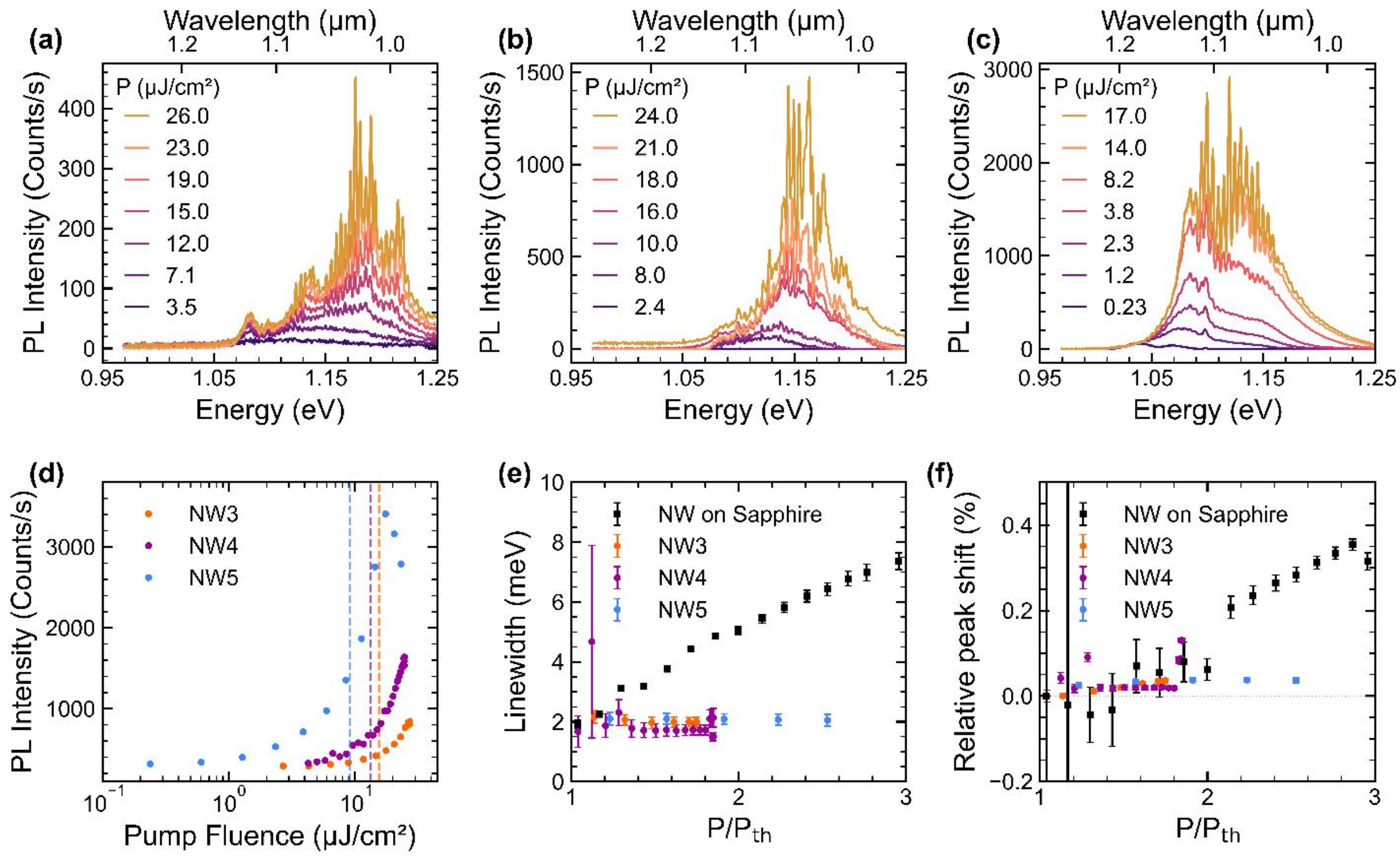

Figure S5: Statistical fluence-dependent lasing characteristics of resonator-integrated nanowires. (a–c) Pump-fluence dependent μ-PL spectra of three additional GaAsSb nanowires (NW3–NW5) transfer-printed onto silicon racetrack resonators. (d) Corresponding input–output characteristics indicating lasing thresholds in the range of 9.2 – 15.5 μJ/cm². (e) Emission linewidth evolution above threshold compared to the standalone nanowire on sapphire. (f) Relative shift of the dominant lasing peak above threshold, demonstrating strongly improved wavelength stability for resonator-coupled nanowires.

Figure S5 provides additional device statistics demonstrating that the lasing characteristics reported in the main text are reproducibly obtained across multiple transfer-printed GaAsSb nanowires integrated on silicon racetrack resonators. The measurements were performed using a Spectra-Physics Tsunami Ti:sapphire excitation laser, a Mitutoyo Apo Plan 50X objective, PiezoJena x-y-z nanopositioners, a helium flow cryostat, a HORIBA Triax grating spectrometer, and a liquid-nitrogen-cooled 1024-pixel InGaAs array detector. Panels **(Figure S5**a–c**)** show pump-fluence dependent μ-PL spectra for three further devices (NW3–NW5), each aligned and

placed onto the straight arm of the racetrack resonator using the deterministic transfer-printing approach described in **Figure S1**b. For all nanowires, a clear transition from broadband spontaneous emission to narrow, sharply defined spectral features is observed upon increasing excitation fluence, consistent with the onset of laser oscillation. The corresponding input–output characteristics are summarized in **Figure S5**d. All three devices exhibit pronounced threshold behavior, confirming lasing action across the sample set. Extracted lasing thresholds range from 9.2 to 15.5 μJ/cm², reflecting moderate device-to-device variations that are expected due to small differences in nanowire diameterand positioning. Beyond threshold, the linewidth evolution shown in **Figure S5**e further highlights the stabilizing role of the passive silicon cavity. While the standalone reference nanowire on sapphire exhibits strong linewidth broadening with increasing pump fluence, all resonator-integrated devices maintain a nearly constant linewidth of approximately 2 meV over the investigated excitation range. This behavior indicates that the dominant lasing mode remains spectrally well-defined even at elevated carrier densities, in stark contrast to the strong broadening typically observed for nanowire Fabry–Pérot cavities where the active medium itself defines the resonator. Consistently, the enhanced frequency stability is further quantified in **Figure S5**f, which plots the relative shift of the dominant lasing peak above threshold with respect to its spectral position at $P=P_{th}$. For all three resonator-coupled nanowires, the peak position remains remarkably stable, with relative shifts below 0.05%, even at high pump levels. This is significantly smaller than the ∼0.4% shift observed for the standalone nanowire on sapphire under comparable excitation conditions. The improved stability arises because the effective cavity is largely determined by the passive silicon racetrack resonator, thereby strongly reducing sensitivity to carrier-induced refractive index changes within the nanowire gain medium.

Together, the statistical analysis confirms that resonator integration consistently yields nanowire lasers with improved spectral robustness, demonstrating the reproducibility of the hybrid NW-resonator platform.

References

[1] D. Jevtics et al., "Characterization, Selection, and Microassembly of Nanowire Laser Systems," Nano Letters, vol. 20, no. 3, pp. 1862–1868, 2020, doi: 10.1021/acs.nanolett.9b05078

[2] J. B. Khurgin and M. A. Noginov, "How Do the Purcell Factor, the Q-Factor, and the Beta Factor Affect the Laser Threshold?," *Laser & Photonics Reviews*, vol. 15, no. 3, 2021, doi: 10.1002/lpor.202000250
